# Sympatric Speciation in Mole Rats and Wild Barley and Their Genome Repeatome Evolution: A Commentary

**DOI:** 10.1002/ggn2.202200009

**Published:** 2022-08-25

**Authors:** Eviatar Nevo, Kexin Li

**Affiliations:** ^1^ Institute of Evolution University of Haifa Haifa 3498838 Israel; ^2^ State Key Laboratory of Grassland Agro‐ecosystem College of Ecology Lanzhou University Lanzhou 730000 China

**Keywords:** “Evolution Canyon”, “Evolution Plateau”, coding and noncoding genomes, genome mirroring, repeatome, subterranean mole rats, sympatric speciation, wild barley

## Abstract

The theories of sympatric speciation (SS) and coding and noncoding (cd and ncd =repeatome)  genome function are still contentious. Studies on SS in our two new models, “Evolution Canyon” and “Evolution Plateau”, in Israel, divergent microclimatically and geologically‐edaphically, respectively, indicated that in ecologically divergent microsites SS is a common speciation model across life from bacteria to mammals. Genomically, the intergenic ncd repeatome was and is still regarded by many biologists as “selfish,” “junk,” and non‐functional. In contrast, it is considered by the encyclopedia of DNA elements discovery as biochemically functional and regulatory, and the transposable elements were considered earlier by Barbara McClintock as “controlling elements” of genes. Remarkably, it is found that repeated elements can statistically identify significantly, the five species of subterranean mole rats of *Spalax ehrenbergi* superspecies adapted to increasingly arid climatic trend southward in Israel. Moreover, it is first discovered in the SS studies in two distant taxa, subterranean mole rats and wild barley, and later also in spiny mice in Israel and subterranean zokors in China, that the noncoding repeatome is genomically mirroring the image of the protein‐coding genome in divergent ecologies. It is shown that this mirroring image is statistically significant both within and between the ecologically divergent taxa supporting the hypothesis that much of the repeatome might be regulatory and selected as the protein‐coding genome by the same ecological stresses.

## Graphical abstract

Our discovery in subterranean mammals and wild barley that noncoding genome or repeatome shows a mirror image of the coding genome suggests that they are both selected by the same ecological stresses and are both functionally adaptive.

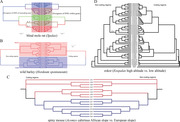

## 1 Introduction

SS, first suggested by Darwin,^[^ ^1^ ^]^ though phrased differently, remains controversial.  Our last 30 years' studies on the role of SS in evolution^[^ ^2^ ^]^ suggest that SS appears to be a common model in nature. SS requires extreme habitat or environmental differences that are within individual dispersal distances.^[^ ^3^ ^]^ and there are numerous “Evolution Canyons” and “Evolution Plateaus”, that is, microsites divergent ecologically across the planet with primary SS^[^ ^4^ ^]^ and pre‐plus post‐zygotic reproductive isolation mechanisms^[^ ^5^ ^]^ in abutting taxa. They involve climatic, geologic, edaphic, abiotic, and biotic ecological contrasts causing SS globally from viruses and bacteria to fungi, plants, and animals.^[^ ^2^ ^]^ Our SS genomic studies revealed dramatic results concerning the coding and noncoding genomes in two of our SS model organisms: blind subterranean mole rats, *Spalax ehrenbergi* superspecies^[^ ^6^ ^]^ in Israel, and wild barley, *Hordeum spontaneum*,^[^ ^4^ ^]^ the progenitor of cultivated barley, both in eastern upper Galilee, Israel in “Evolution Plateau”, described below.

The structure and function of the genome have been and are still hotly debated. The idea of protein‐coding versus noncoding “junk” genome^[^ ^7^ ^]^ (and others detailed in the discussion) was transferred with the encyclopedia of DNA elements (ENCODE) highlights (Consortium 2012).^[^ ^8^ ^]^ ENCODE revealed that the human genome is copied into RNA, and interactive regulatory proteins, organizing the genome into chromatin and cellular divergence. Remarkably, ENCODE disclosed that up to 80% of the human genome contained biochemically functional elements invalidating the view that the human genome consists mostly of “Junk DNA” and “selfish genes”. In contrast, intergenic spaces are filled with DNA regulatory elements, enhancers, promoters, and RNA transcripts that are not translated into proteins but might have a regulatory role.^[^ ^9^ ^]^ Regulation, rather than “junk” and “selfishness,” might be the main role of much of the noncoding and repetitive DNA. The discovery of Britten and Khone of mobile and repetitive DNA,^[^ ^10^ ^]^ and the discovery of Barbara McClintock^[^ ^11^ ^]^ that mobile elements may be “controlling elements” of genes in maize,^[^ ^11^ ^]^ negated and transformed the “junk” genome hypothesis into the largely regulatory noncoding genome. The debate between “junk DNA” and the regulatory noncoding genome is ongoing but seems to be largely transformed into a regulatory genome with solid evidence.^[^ ^12^ ^]^ It is noteworthy that, James Shapiro had a long‐lasting interest in the repeatome since up to two‐thirds of the human genome is composed of mobile genetic elements, and he first discovered them in bacteria and suggested that these elements do not behave in their host as selfish DNA but as a co‐operative component for the evolution of bacteria.^[^ ^13^ ^]^ Shapiro's major article with Sterenberg was titled “Why repetitive DNA is essential to genome function”.^[^ ^14^ ^]^ This article anticipated the recent ENCODE results. Its basic idea was that the genome is a highly sophisticated information storage organelle, highly formatted by generic (i.e., repeated) signals enabling it to access the stored information, when and where it will be useful (see ref. [12]). The main message indicates that there are clear theoretical reasons and examples showing that repetitive DNA has an essential genome function^[^ ^14^ ^].^ Following are our SS results in subterranean mammals, first in the *S. ehrenbergi* superspecies and second in wild barley, *H. spontaneum*, bearing on the controversial issue of genome structure and evolution.

## 2 Highlighting Mirroring of Coding and Noncoding Genomes in Subterranean Mole Rats

**Figure 1:**
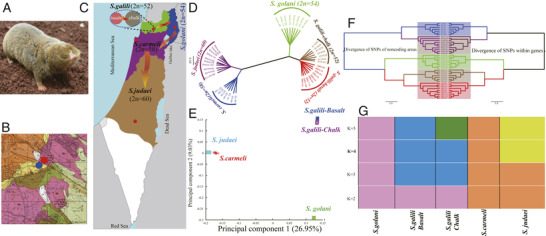
The ecogeographic distribution and the genomic divergence among *Spalax* species in Israel (From Ref. [15]). A) Blind mole rat, *Spalax*. B) Geological map of east Upper Galilee including Evolution plateau and the two sympatric species colored blue, *Spalax galili* chalk (2*n* = 52), the progenitor, and red the derivative new sympatric species (2*n* = 52). C) Ecogeographic map of *Spalax ehrenbergi* superspecies distribution (from north to the south marked in different colors) and sampling sites (red dots) of the four climatic and chromosomal peripatric species and the fifth *S. galili* (2*n* = 52) marked in green, which diverged geologically—edaphically, genically (not chromosomally), sympatric species derivative *S. galili* basalt (2*n* = 52); *S. golani* (2*n* = 54) marked in blue, *S. carmeli* (2*n* = 58) marked in violet, *S. judaei* (2*n* = 60) marked in brown. D) A neighbor‐joining tree was reconstructed with the allele shared matrix of SNPs of the five blind mole rat species populations, and the scale bar represents the *p* distance. E) Genetic clusters of the four species showed by PCA based on SNPs, only principal component 1 (26.95%) and principal component 2 (9.03%) are displayed. F) Neighbor‐joining tree based on the SNPs located in the remarkably mirroring coding and noncoding genomes, respectively. G) Structure: genetic bar plots of the five *Spalax* species. The number of putatively genetic populations (*K*) was defined from *K* = 2 to *K* = 5, each column denotes one individual. Reproduced with permission.^[^ ^15^ ^]^ Copyright 2020, PNAS.

**Figure 2:**
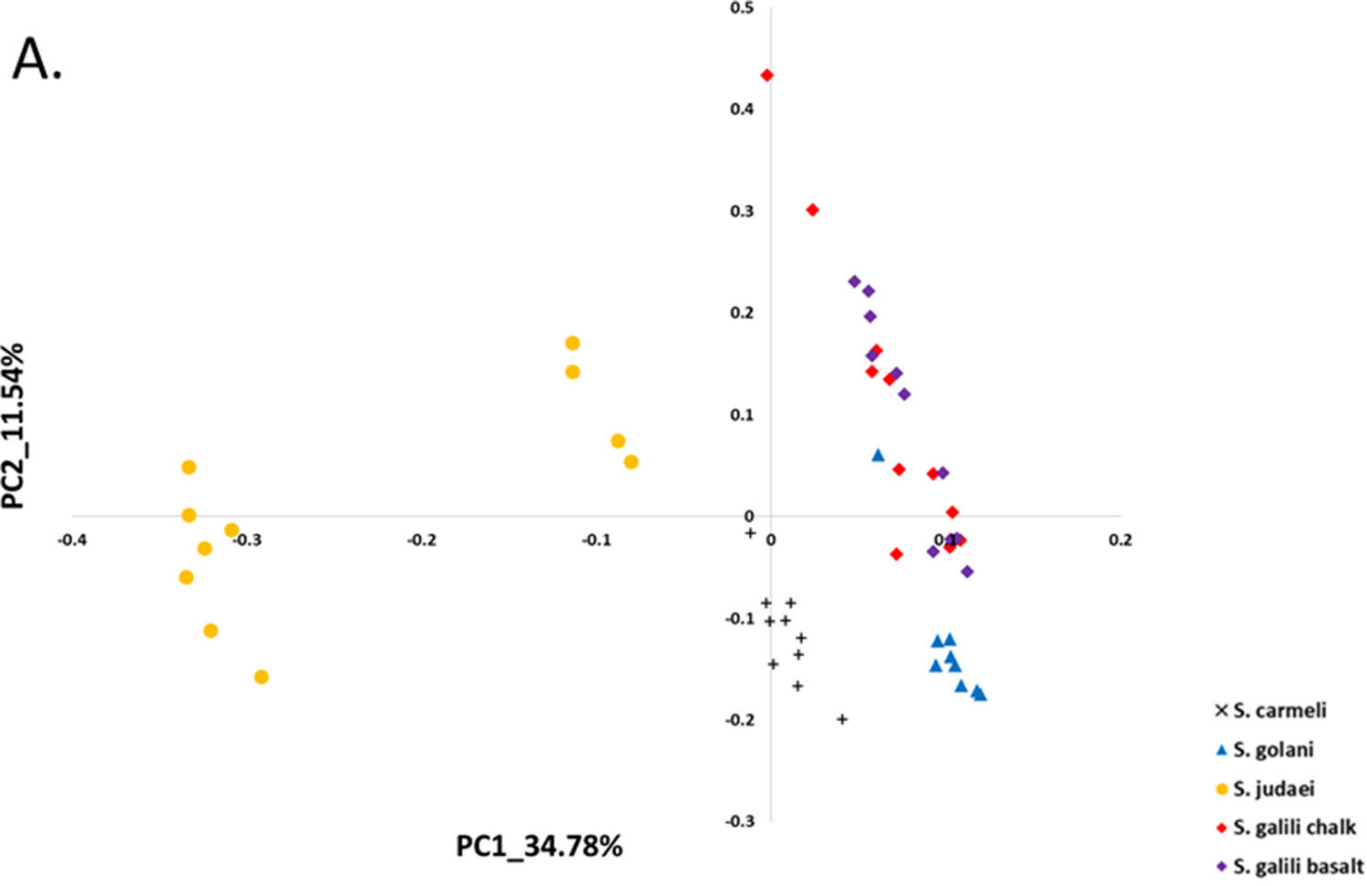
The 2D plot shows the projection of abundance data of well‐annotated 300 RepBase repetitive elements on the first two PCs. The samples are colored according to *S. ehrenbergi* superspecies. Note that the four chromosomal species are all separated by the repetitive elements, whereas the sympatric pair *S. galili* chalk (ancestor) and *S. galili* basalt (derivative) are overlapping, unlike the four chromosomal species (but see Figure 10 where also this sympatric pair can be separated by repeated elements by the Random Forest analysis (Figure S15A, Supporting Information). Reproduced with permission.^[^ ^15^ ^]^ Copyright 2020, PNAS.

**Figure 3:**
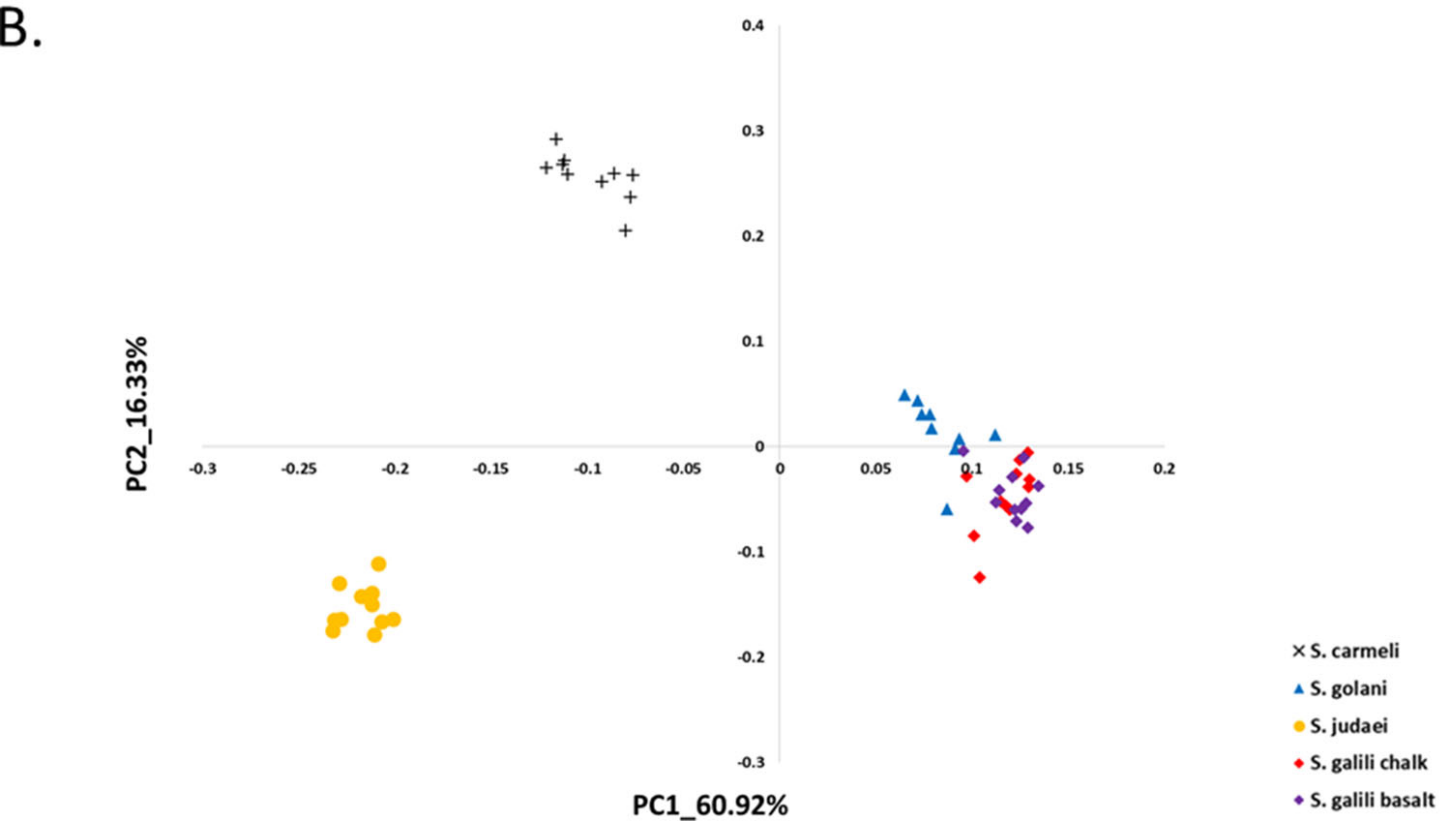
The 2D plot shows the projection of k‐Chain abundance data of the *S. ehrenbergi* superspecies in Israel based on the first two PCs. The samples are colored according to *S. ehrenbergi* superspecies.): *S. carmeli*, 2*n =* 58; *S. golani*, 2*n* = 54; *S. judaei*, 2*n* = 60. *S. galili* chalk and basalt, 2n = 52. Note the separation of 2n = 54, 58, 60, and only partial separation of *S. galili* complex chalk and basalt. Possibly PC 3 could also separate the *S. galili* sympatric complex (From Ref. [15], Figure S15B, Supporting Information**)**. Reproduced with permission.^[^ ^15^ ^]^ Copyright 2020, PNAS.

**Figure 4:**
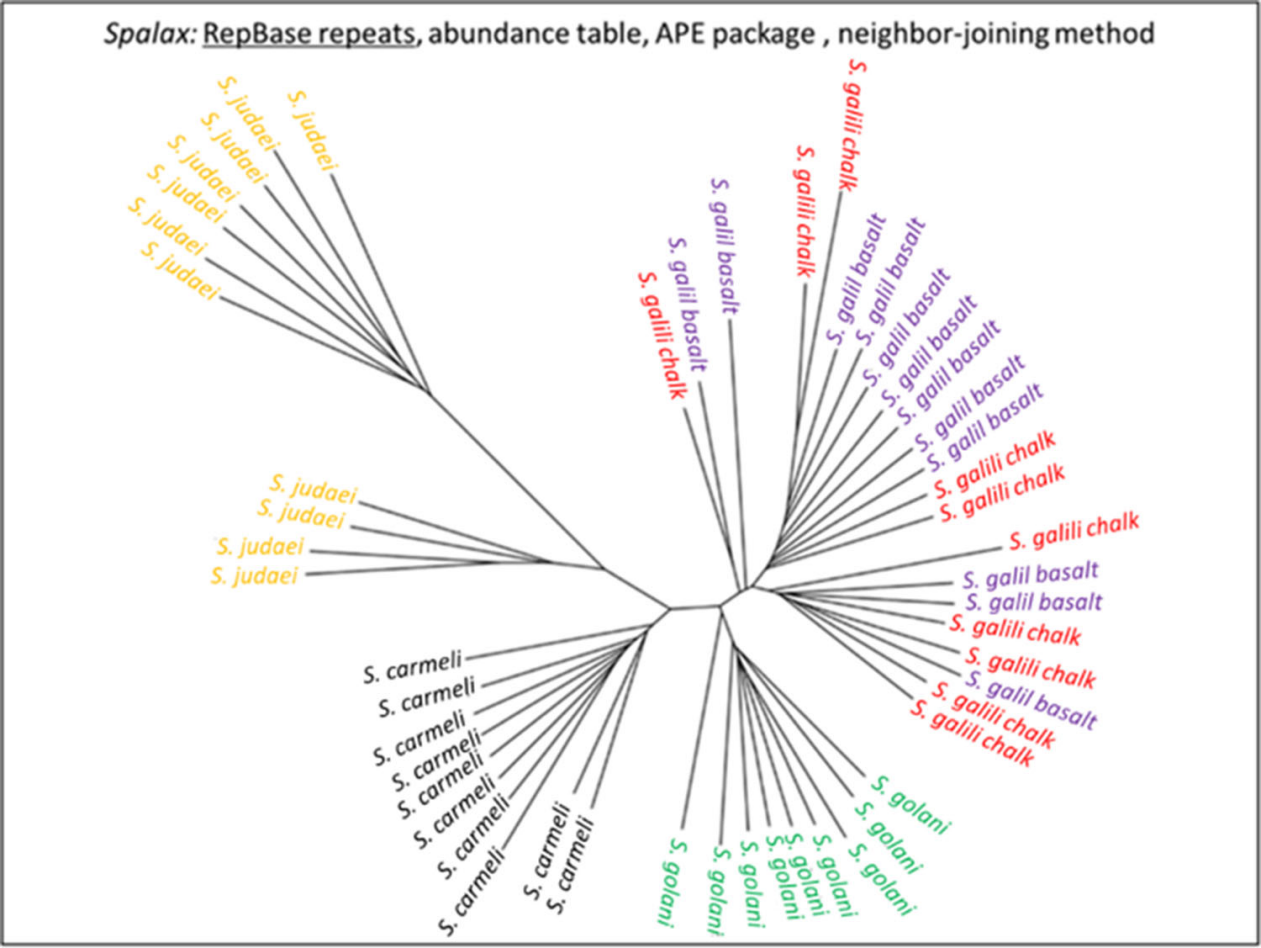
Unrooted phylogenetic tree of the Israeli *S. ehrenbergi* superspecies by repeatome, with only partial separation of the *S. galili*, 2*n =* 52 sympatric complex, based on the abundance of RepBase annotated repetitive elements. The tree was prepared with Ape package with a neighbor‐joining method Note the separation of 2*n* = 54, 58, 60, and only partial separation of the *S. galili* sympatric complex. (From Ref. [15], Figure S15C, Supporting Information). Reproduced with permission.^[^ ^15^ ^]^ Copyright 2020, PNAS.

**Figure 5:**
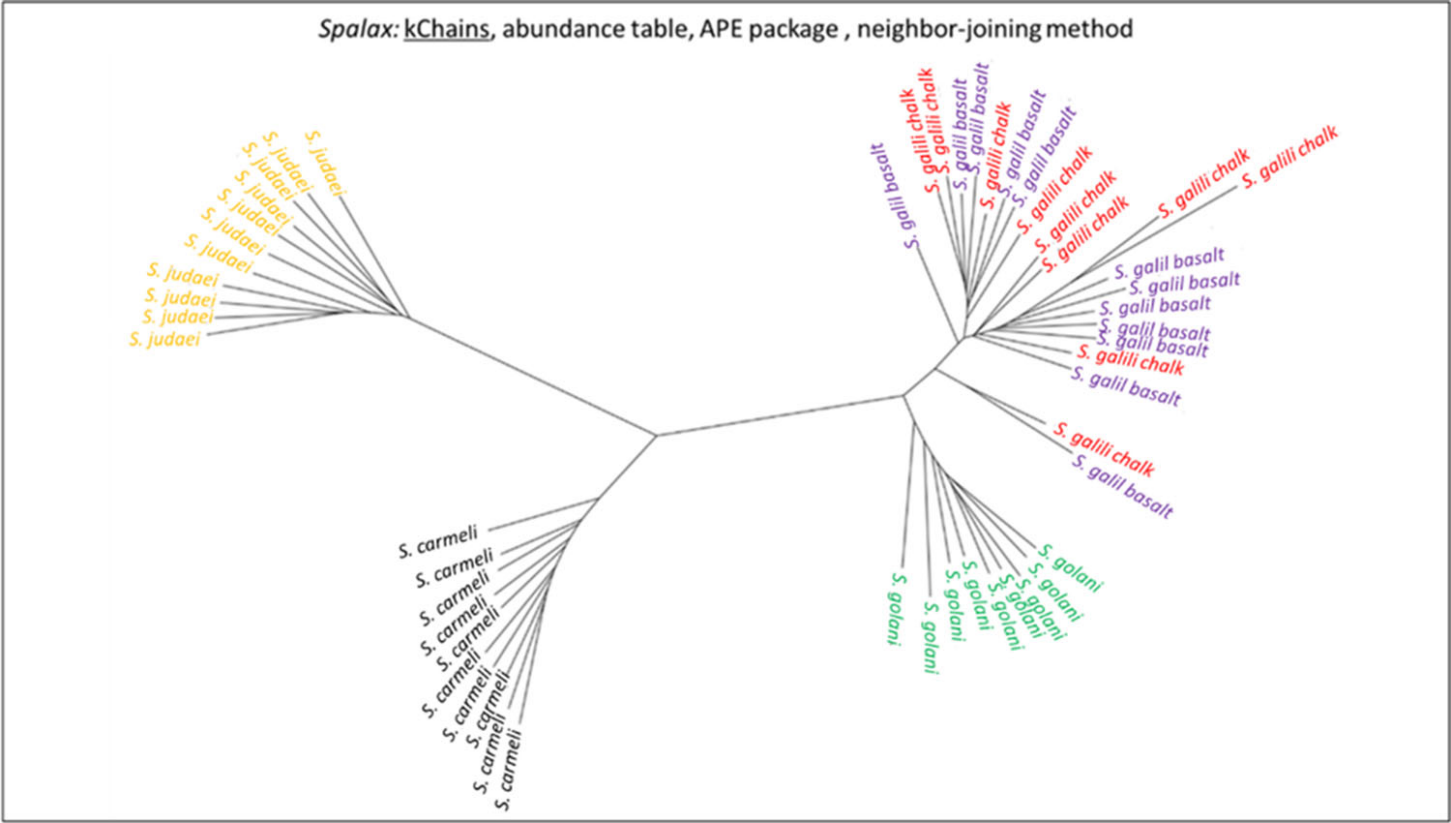
Unrooted phylogenetic tree based on the abundance of k‐Chains. The tree was prepared with Ape package with neighbor‐joining method (From Ref. [15], Figure S15D, Supporting Information). Reproduced with permission.^[^ ^15^ ^]^ Copyright 2020, PNAS.

**Figure 6:**
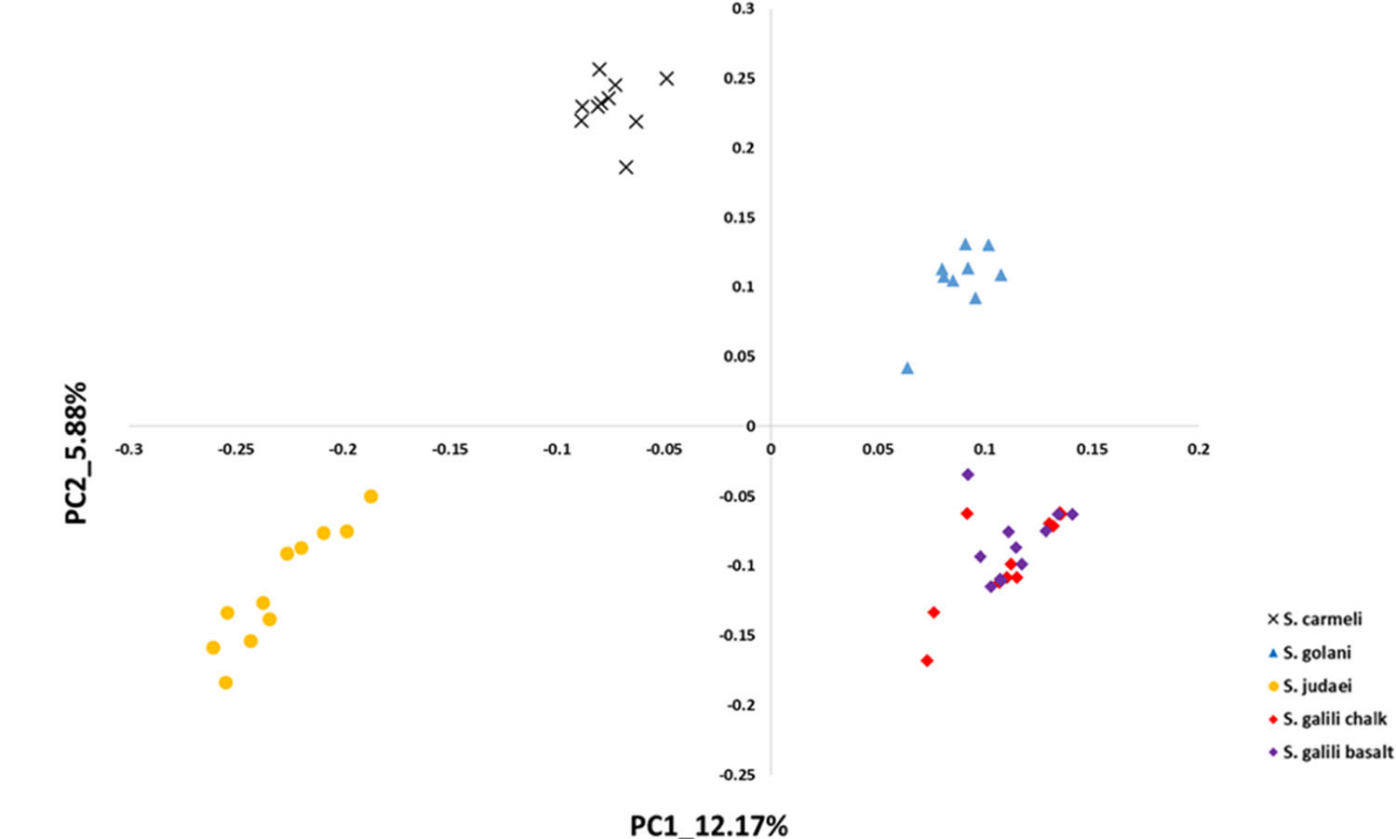
The 2D plot shows the projection of mutability data of well annotated 300 RepBase repetitive elements on the first two PCs. The samples are colored according to *S. ehrenbergi* superspecies (From Ref. [15], Figure S16A, Supporting Information). Reproduced with permission.^[^ ^15^ ^]^ Copyright 2020, PNAS.

**Figure 7:**
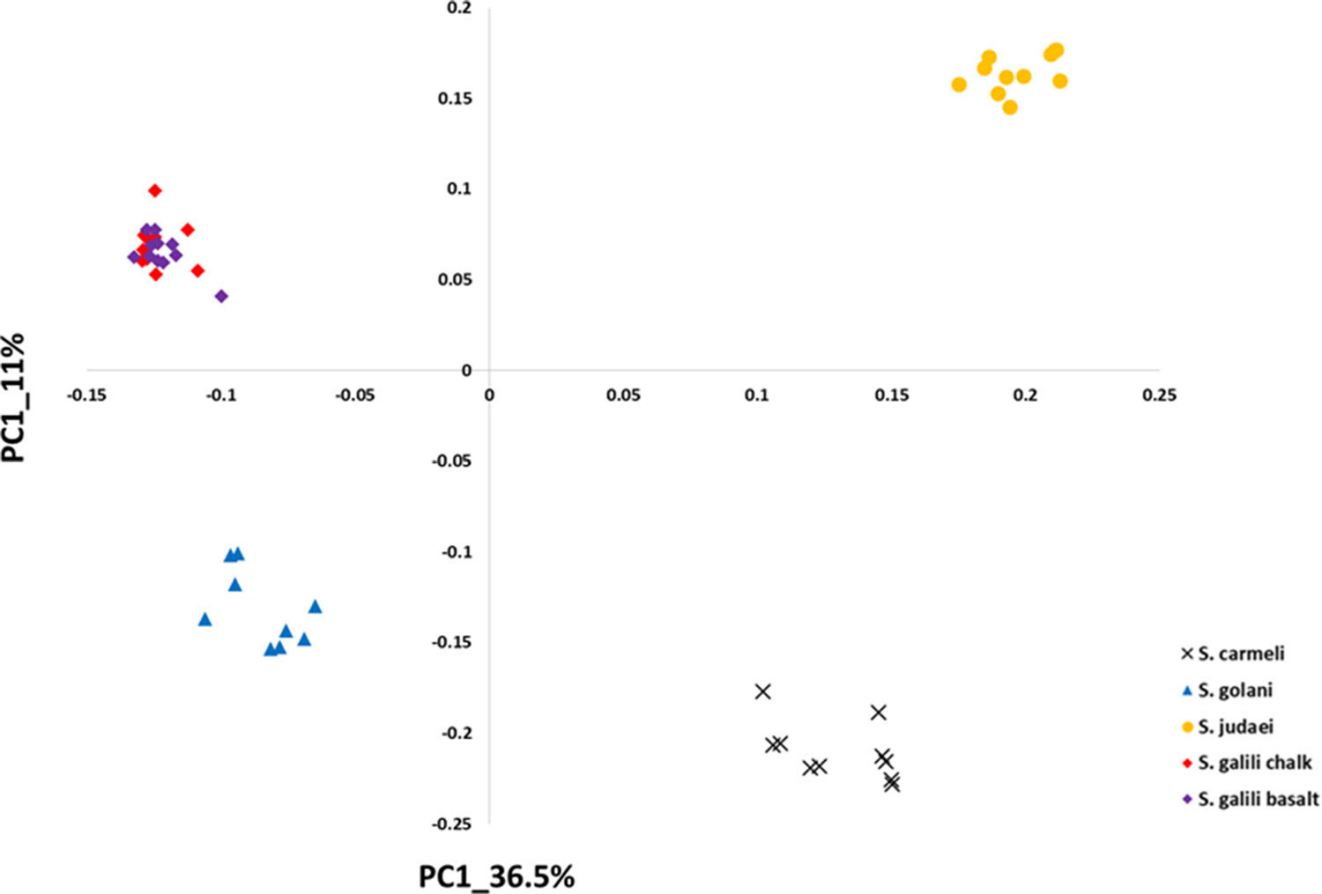
The 2D plot shows the projection of k‐Chain mutability data on the first two PCs. The samples are colored according to *S. ehrenbergi* superspecies. (From Ref. [15] Figure S16B, Supporting Information). Reproduced with permission.^[^ ^15^ ^]^ Copyright 2020, PNAS.

**Figure 8:**
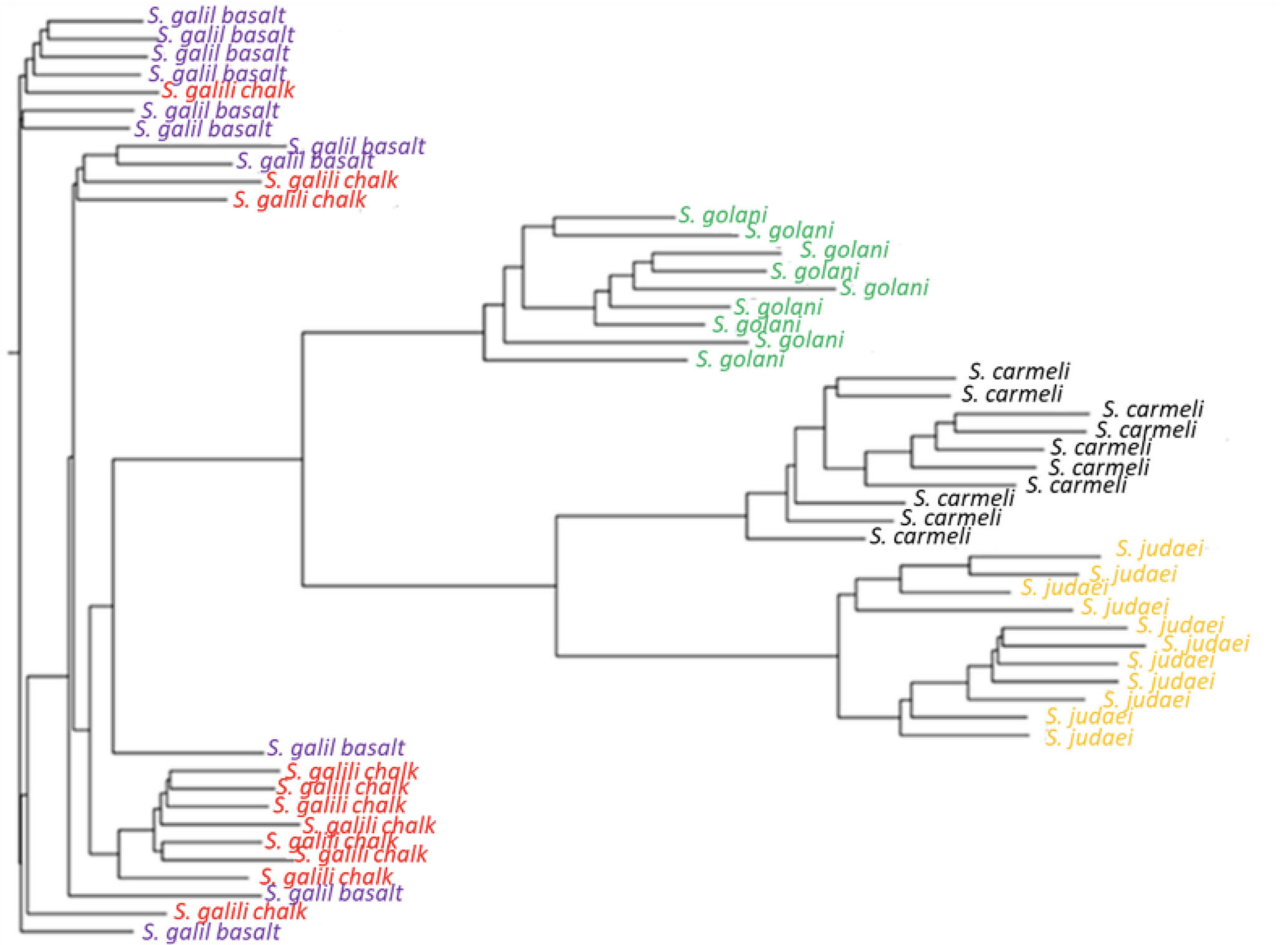
Unrooted phylogenetic tree prepared with Ape package based on mutability of k‐Chains. Note that *S. galili* chalk basalt is largely separated. (From Ref. [15], Figure S16B, Supporting Information). Reproduced with permission,^[^ ^15^ ^]^ Copyright 2020, PNAS.

**Figure 9:**
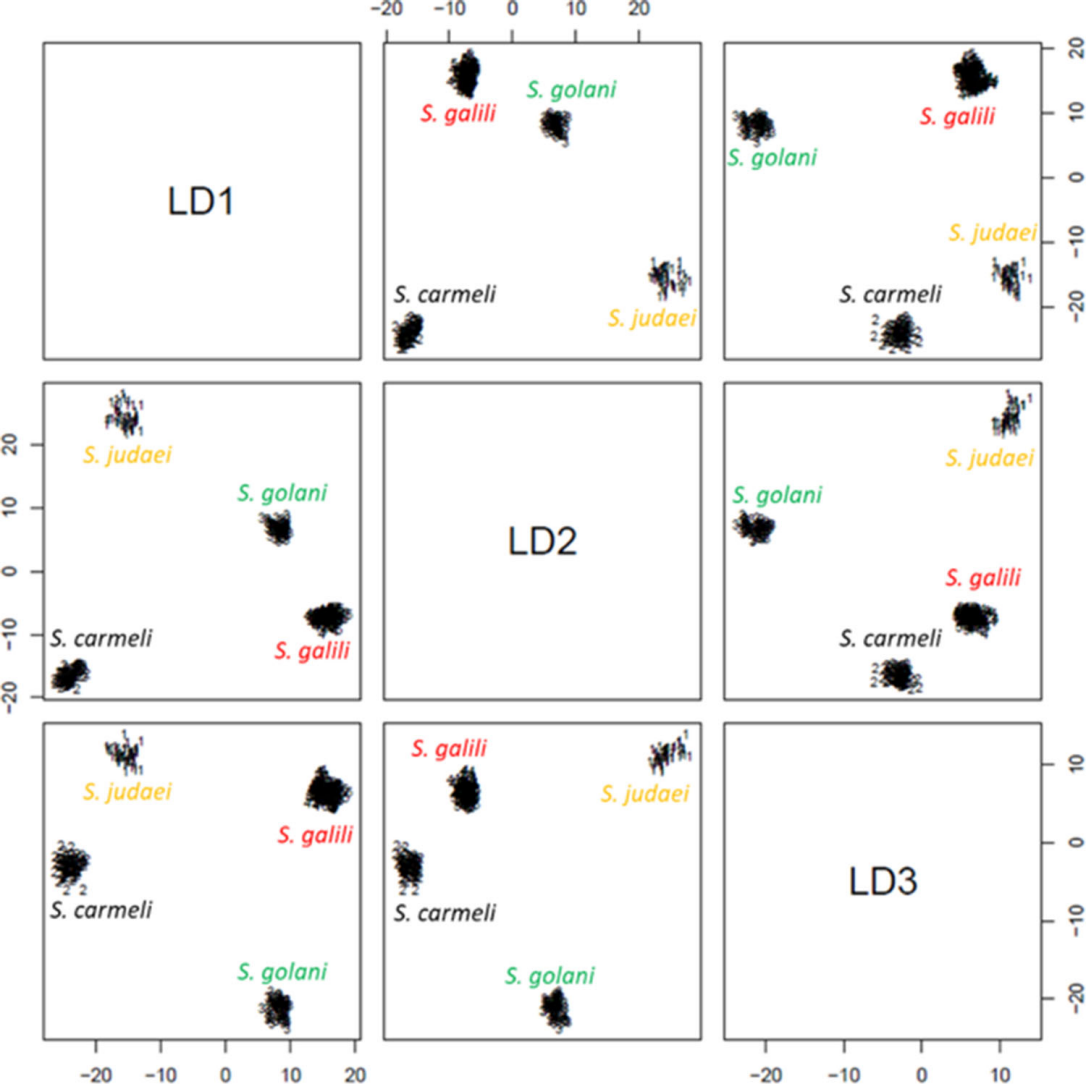
Putative repetitive elements (k‐Chains) identified as differentiating between *S. ehrenbergi* superspecies four major chromosomal species (From Ref. [15], Figure S17A, Supporting Information). Reproduced with permission.^[^ ^15^ ^]^ Copyright 2020, PNAS.

**Figure 10:**
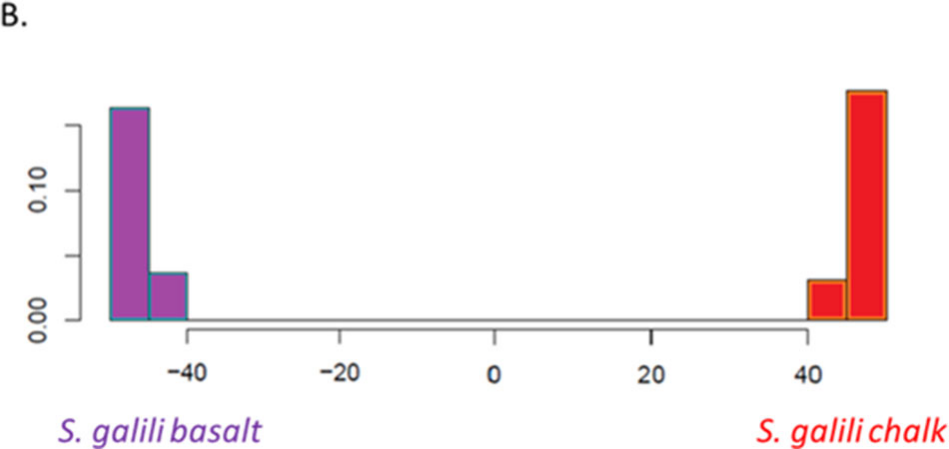
The 2D plot shows that the LD1‐LD2 projection of an abundance of 150 k‐Chains differentiated between two *S. galili* sympatric species (Chalk ancestor and basalt derivative) by Random Forest analysis.) Importantly, Random Forest analysis can even differentiate between the chalk progenitor and the basalt new derivative species that originated by sympatric speciation 228 000 years ago (From Ref. [15], Figure S17 B, Supporting Information). Reproduced with permission.^[^ ^15^ ^]^ Copyright 2020, PNAS.

**Figure 11:**
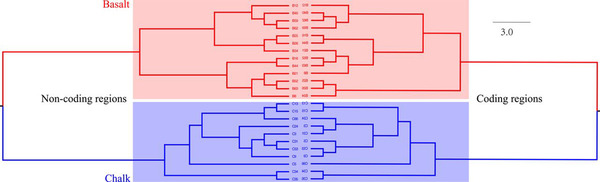
Neighbor‐joining tree of wild barley, *Hordeum spontaneum*, from “Evolution Plateau”, Upper Galilee Israel, the progenitor of cultivated barley, *Hordeum vulgare*, based on the SNPs from coding and noncoding genomic regions. The numbers in the middle indicate the genotypes studied in each soil population. Note the mirror image of the coding and noncoding genomes demonstrating that both are subjected to the same ecological stresses and are selected accordingly. Therefore, the noncoding genome cannot be either “Junk” or “selfish” DNA” but appears to be regulatory as in mole rat *Spalax* described above (From Ref. [4], Figure S2, Supporting Information). Reproduced with permission.^[^ ^4^ ^]^ Copyright 2020, Life Science Alliance.

Speciation mechanisms remain controversial. Two speciation models occur in Israeli subterranean mole rats, genus Spalax: a regional speciation cline southward of four peripatric, climatic, chromosomal species and a local, geologic‐edaphic, genic, and SS complex (**Figure** **1**A–G). In ref. [15], we highlighted mole rat genome evolution. The five species we discovered in Israel and studied genomically were separated into five genetic clusters by single nucleotide polymorphisms, copy number variations (CNVs), repeatome, and methylome in sympatry (Figure 1A–E, **Figures** **2**, **3**, **4**, **5**, **6**, **7**, **8**, **9**, **10**). The regional interspecific divergence corresponds to Pleistocene climatic cycles. Climate warmings caused chromosomal speciation in four species: *Spalax golani*, 2*n* = 54; *Spalax galili*, 2n = 52; *Spalax carmeli*, 2n = 58, *Spalax judaei*; and in one sympatric species in the *S. galili* complex, where the sympatrically originated species in *S. galili* (basalt) originated from *S. galili* (chalk), (temporary names), both ancestor and derivative species consist of 2*n* = 52 and are divergent geologically by abutting Senonian Chalk and Pleistocene basalt.^[^ ^6^, ^16^, ^17^, ^18^, ^19^, ^20^, ^21^ ^]^ Triple effective population size, Ne, declines match glacial cold cycles. Adaptive genes evolved under positive selection to underground stresses and to divergent climates, involving interspecies reproductive isolation. Genomic islands evolved mainly due to adaptive evolution involving ancient polymorphisms. Remarkably, repeatome, including both CNV and LINE1 repetitive elements, separated the five species (Figures 1, 2, 3, 4, 5, 6, 7, 8, 9, 10). Methylation in sympatry also identified the geologically chalk‐basalt species that differentially affect thermoregulation, hypoxia, DNA repair, P53, and other pathways. Genome adaptive evolution highlights climatic and geologic‐edaphic stress evolution and the two speciation models, peripatric and sympatric. Remarkably, the noncoding genome mirrors completely the coding genome (Figure 1F), both in subterranean mole rats in the five Israeli *Spalax* species and wild barley, *H. spontaneum*, from the same microsite of “Evolution Plateau” (**Figure** **11**). The mirror image of the coding and noncoding genomes clearly indicates that both genome regions are selected equally by the same ecological stresses, hence suggesting their biochemical functionality not only in humans but first also in other eukaryotes (Figures 1F, Figures 2, 3, 4, 5, 6, 7, 8, 9, 10).

## 3 Highlighting Mirroring of the Coding and Noncoding Genomes in Wild Barley

**Figure 12:**
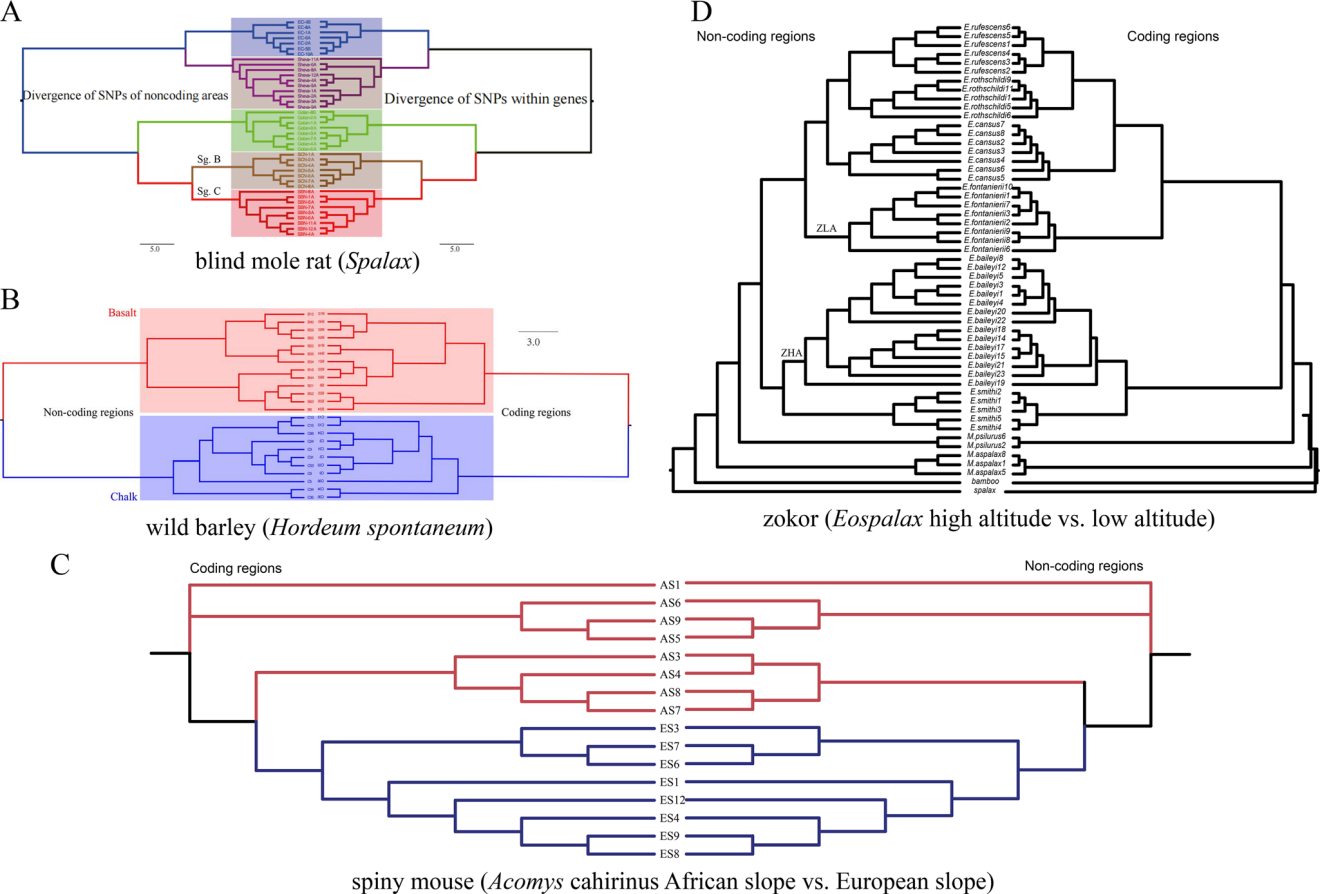
Phylogeny of A) blind mole rat, *S. galili*, B) wild barley, *H. spontaneum*, C) spiny mouse, *Acomys cahirinus*, and D) zokor, *Eospalax fontanieri* based on SNPs from genes and intergenic regions.^[^ ^4^, ^15^, ^22^, ^23^ ^]^

SS has been contentious since the idea was suggested by Darwin. Here, we show in wild barley SS due to geologic and edaphic divergence in “Evolution Plateau”, Upper Galilee, Israel (4). Our whole‐genome resequencing data showed SS separating between the progenitor old Senonian chalk and abutting derivative young Pleistocene basalt wild barley populations. The basalt wild barley species unfolds larger effective population size, lower recombination rates, and larger genetic diversity. Both species populations show a similar descending trend ≈200 000 years ago associated with the last glacial maximum. Coalescent demography analysis indicates that SS was local, primary, in situ, and not due to a secondary contact from an ex‐situ allopatric population. Adaptive divergent putatively selected genes were identified in both populations. Remarkably, disease‐resistant genes were selected in the wet basalt population, and genes related to flowering time, leading to temporal reproductive isolation, were selected in the chalk population. The evidence substantiates adaptive ecological SS in wild barley, highlighting the genome landscape during SS with gene flow, due to geologic‐edaphic divergence. The dramatic discovery is that in both *Spalax* species (Figure 1, 2, 3, 4, 5, 6, 7, 8, 9, 10) and wild barley at the “Evolution Plateau” noncoding genome mirrors the coding genome (Figure 11), suggesting that, like in *Spalax* described earlier, they are responding to the same ecological stresses, and that it might be found in other eukaryotes (**Figure** **12**).

**Figure 13:**
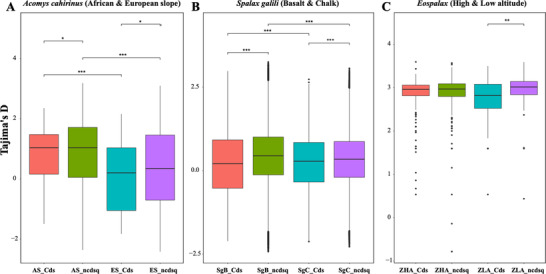
Differences in Tajima's D between the selected and nonselected genome regions in three taxa. A) Differences between *A. cahirinus* from the African slope (in red, *n* = 8) at Evolution Canyon, Mount Carmel, Israel, and the opposite European slope in green (*n* = 8). Cds = coding sequences, ncdsq = noncoding sequences. B) The differences between *S. galili* chalk (*n* = 6) and basalt (n = 9), Chalk = SC, Basalt = SB. Chalk is the ancestor species and Basalt is the derivative sympatric species, both in Upper Galilee Israel. C) Comparison of Tajima's D between high altitude (*n* = 9) and low altitude (*n* = 7) zokors. Statistical significances were showed in the figure for all three taxa.

In order to check whether noncoding regions were under the same selection like that in coding regions, we identified all the selected regions across the genome. In total, 21.5 Mb sequences were under selection, including 13.5 Mb (0.9% of the total noncoding regions) from noncoding regions and 8 Mb (1.03% of the total gene regions) from coding regions. We compared the Tajima's *D* between selected genes and non‐coding regions and found significant differences in *Acomys* (**Figure** **13**A) and significant in *Spalax* (Figure 13B).

We compared the difference of Tajima's *D* for *A. cahirinus* from African slope of Evolution Canyon. Mount Carmel, Israel (sample size (*n*) = 8) and European slope (*n* = 8), *S. galili* inhabited in Evolution Plateau, eastern Upper Galilee, the following: basaltic soil (*n* = 9) and chalk soil (*n* = 6). In *Eospalax* from high altitude (*n* = 9) and low altitude (*n* = 7) of Qinghai‐Tibet Plateau in China. For each group, the whole‐genome SNPs were divided into two sections, the coding genes SNP sequences (CDs) and noncoding SNP sequences (non‐CDs), respectively. Tajima's *D* was calculated using VCFtools v0.1.16 for every 50 kb sliding window. Outliers were eliminated with the median absolute deviation (MAD) algorithm. The mean Tajima's *D* for *A. cahirinus* from the African slope were 0.801 ± 0.168 (non‐CDs) and 0.618 ± 0.849 (CDs), and European were 0.269 ± 1.259 (non‐CDs) and 0.345 ± 0.899 (CDs). The mean Tajima's D for *S. galili* in basalt were 0.239 ± 0.895 (CDs) and 0.432 ± 0.823 (non‐CDs), and chalk were 0.194 ± 0.884 (CDs) and 0.335 ± 0.783 (non‐CDs). The mean Tajima's D for *Eospalax* from high altitudes were 2.847 ± 0.446 (CDs) and 2.882 ± 0.453 (non‐CDs), and at low altitudes were 2.681 ± 0.608 (CDs) and 2.957 ± 0.378 (non‐CDs). The box plots for the rest of Tajima's *D* values were drawn with the R package “ggplot2”, and the significant difference analysis for different regions or groups was performed by Wilcoxon Rank Test (*(*p* < 0.05); **(*p* < 0.01); ***(*p* < 0.001)). The highest and lowest spots for each box plot represent the maximum and minimum Tajima’ s *D*, and strigulas from bottom to top represent the first quartile, median and third quartile of Tajima’ s *D*, respectively.

## 4 Discussion

Britten RJ and Kohne, D.^[^ ^10^ ^]^ discovered repeated sequences in human DNA. Hundreds of thousands of copies of DNA sequences have been incorporated into the genomes of higher organisms. These intergenic repeats have been described as both “junk and selfish” by,^[^ ^7^ ^]^ and are still believed to be “junk and selfish” by many genome researchers. Remarkably, however, ENCODE (Consortium, 2012)^[^ ^8^ ^]^ identified in humans 80% biochemically functional regulatory DNA elements by an extensive and intensive research program.^[^ ^8^ ^]^ However, their functionality has been questioned.^[^ ^24^, ^25^, ^26^, ^27^, ^28^ ^]^ Our interdisciplinary evolutionary studies in *S. ehrenbergi* superspecies since 1948, and on wild barley, and wild emmer wheat since 1975, clearly demonstrate adaptive evolution and SS in all these three species affecting their phenotypes and genotype evolution [See revisit of our studies in two models, Evolution Canyon with four repeats in the Carmel, Galilee, Golan, and Negev Mountains, and Evolution Plateau in upper Galilee revisited in Nevo, 2021,^[^ ^2^ ^]^ and the full lists of subterranean mole rats, *S. ehrenbrgi* superspecies, and wild cereals of Nevo at http://evolution.haifa.ac.il]). We studied genetic diversity in multiple natural populations (e.g., refs. [30, 32], revealed that genetic diversity in nature, is structured, nonrandom, and can be largely explained by ecological stresses, including primarily climatic, geologic‐edaphic, abiotic, and biotic factors involving parasites and diseases). We rejected neutrality evolution theory as driving evolution^[^ ^31^, ^32^ ^]^ (Figure 13). Our studies revealed that stress evolution, primarily by ecological factors, appears to drive adaptive evolution.^[^ ^2^ ^]^ Our discovery here in subterranean mammals of the *S. ehrenbergi* superspecies across Israel, and in wild barley *H. spontaneum* at Evolution Plateau summarized above, plus spiny mouse *Acomys cahirirus* ^[^ ^22^ ^]^ and zokors subterranean mammals in China,^[^ ^23^ ^]^ stunningly suggests that the repeatome, comprising the extensive region of the genome, mirrors equally the much smaller coding genome (Figures 1, 2, 3, 4, 5, 6, 7, 8, 9, 10, 11, 12), is significant statistically (Fig. 13); and anticipatingly also in other pro‐and eukaryotes. Our studies strongly support the idea by evidence described above that the repeatome is subjected to ecological stresses driving those of the coding genome. They highlight the substantial importance of the repeatome as the regulator of the coding genome. An early discussion on the potential functionality of the repeated elements has been anticipated by^[^ ^14^ ^]^ and substantiated by the fundamental exciting discovery of ENCODE.^[^ ^8^ ^]^ An earlier discussion on the repeatome and other genome discoveries revealed during the discovery of TEs in bacteria suggested that these elements do not behave in their host as selfish DNA but as a cooperative component for the evolution of bacteria.^[^ ^13^ ^]^ This adaptive hypothesis of the repeatome is reinforced by Shapiro^[^ ^12^ ^]^ and laukien.^[^ ^29^ ^]^

## Conflict of Interest

The authors declare no conflict of interest.
